# Statistical analysis of EBSD data confirms pronounced classical and non-classical pervasive crystallographic twinning in rotaliid foraminiferal calcite

**DOI:** 10.1038/s41598-025-92636-y

**Published:** 2025-04-28

**Authors:** Wolfgang W. Schmahl, X. Yin, J. Lastam, E. Griesshaber, S. Hoerl, E. Sturm, A. Sancho Vaquer

**Affiliations:** 1 Department of Earth and Environmental Sciences, Ludwig Maximilians Universität München, Theresienstraße 41, D-80333 Munich, Germany; 2 Institut für Energie und Klimaforschung, Forschungszentrum Jülich, Wilhelm-Johnen-Straße, 52428 Jülich, Germany; 3 Department: BNSM, Bruker (Beijing) Scientific Technology Co., Ltd, 9F, Building NO.1, Lane 2570, Hechuan Rd, Minhang District, Shanghai, 200233 China

**Keywords:** Calcite twinning, EBSD, Foraminifers, Biomineralization, Amphistegina, Electron microscopy, Marine biology, Biomaterials, Techniques and instrumentation

## Abstract

We describe a quantitative statistical and geometric analysis of classical and non-classical modes of twinning in the calcite produced by biomineralization in the shell of the rotaliid foraminifer species *Amphistegina lessonii.* Foraminifera are responsible for about a quarter of the marine production of CaCO_3_ and thus play a major role in the natural CO_2_ sequestration into marine carbonate sediments. The shell calcite of rotaliid foraminifera is nano-twinned and thus quite distinct from inorganic calcite and from biogenic calcite produced by other groups of organisms. Previous work showed that foraminiferal calcite contains a high spatial density of twin walls of the classical 60°|<001> = m.{001} twin, but there was another peak in the range between 75° and 80° in the misorientation statistics of electron backscatter diffraction (EBSD) maps of the same specimen. We checked the significance of all maxima in misorientation by in-depth statistical analysis, thus confirmed the 60°|<001> penetration twinning and found that the 75°–80° maxima are related to new, non-classical, but systematically reoccurring oriented associations of calcite crystals with orientation relationships 78.2°|<991> and 76.6°|<6 −6 1>. If the nano-twinning provides an evolutionary advantage, it may increase the strength and toughness of the feeble mineralized chamber walls of the organisms.

**Supplementary Information:**

The online version contains supplementary material available at 10.1038/s41598-025-92636-y.

## Introduction

Recently, Yin et al.^1^ and Lastam et al.^2,3^ investigated rotaliid foraminiferal shell calcite with scanning electron microscopy and electron back scatter diffraction (EBSD). They found that foraminiferal calcite - in contrast to other biomineral calcite as well as undeformed geological calcite - is intensively twinned. Most prominent is the twinning by 60° rotation around the crystallographic c-axis ( = <001>), but there are other frequently reoccurring orientational relationships with misorientation angles in the 75°–80° range. The purpose of the present paper is to clarify the observed orientation relationships with a deeper geometrical and statistical analysis with respect to known^4–9^ or potentially newly discovered twin laws, respectively.

The calcite phase of CaCO_3_ is one of the most abundant minerals on the earth’s surface, and marine uni-cellular organisms produce by far most of it. Among them, foraminifera are responsible for about a quarter of the marine production of CaCO_3_ and thus play a major role in the natural CO_2_ sequestration into marine carbonate sediments. Twinning, i.e. intergrowths of crystals of the same crystal phase in a regular, reoccurring fashion, is a common phenomenon in minerals as well as in synthetic crystalline materials. Twinning can have its origin in the loss of symmetry during a structural phase transition, it can be induced by plastic deformation, or it may occur during crystal nucleation and growth. The analysis of calcite twin microstructures is an established tool in the reconstruction of geological conditions of rock formation and rock deformation (e.g^7–9^). Phenomena related to crystal twinning are also of key importance for the physical properties of solid state materials, such as mechanical strength and toughness, piezoelectric effect, shape memory, magnetism, or ferroelectricity, to name but a few. It is common knowledge (see references in^10–15^) that mechanical strength, hardness, and toughness increase if structural defects are present as they impede the motion of dislocations in the crystal structure. Twin walls, notably when closely spaced in the sub-micrometer- range, belong to these structural defects. For example, deformation-induced nano-twinning has been investigated with respect to the increase of strength and enhanced plasticity of metallic materials, thus overcoming the ‘conflict’ between strength and toughness^10–13^. Chen et al.^13^ show how pre-existing nano-twinning (prior to deformation) can be used to increase strength and toughness in engineered materials. As far as biomineralized tissues are concerned, nano-twin-governed toughening in an aragonite-based shell material has been quantitatively demonstrated by Shin et al.^14^, while Li and Ortiz^15^ showed that deformation-induced nano-twinning provides a toughening effect in calcite-based bivalve shell material.

A twin orientation relationship can be expressed by a *twin law*^16^. A twin law is an *orientation relationship* which is frequently a rotation by a specific angle φ around a specific crystallographic axis [*uvw*], or a mirror plane m perpendicular to a specific crystallographic plane normal (*hkl*). However there are also “inversion twins”. Obviously, the same type of twinning can occur with respect to any axis in the symmetry-equivalent set <*uvw*>, or {*hkl*}, respectively. For orientation relationships and twin laws we will use the notations φ|<*uvw*> for rotations and m.{*hkl*} for mirror reflections, respectively. Note that the twin law states the orientation relationship of the two domains/individuals, and it is *not* generally identical to the boundary of intergrowth of the two twin domains.

Inorganic calcite shows four classical twin laws^4–9^. These are the reflection twins with orientation relation (twin law): m.{001}, m.{018}, m.{104}, and m.{012}. The indices we use throughout the paper refer to the hexagonal setting of the calcite unit cell (a_0_ = b_0_ = 4.989Å, c_0_ = 17.062 Å, α = β = 90°, γ = 120°). We describe directions [*uvw*] and plane normals (*hkl*) with three vector components in the basis system defined by these unit cell edges, consistent with the rules of vector arithmetic of contemporary crystallography; four-index Bravais indices will be given in the final result tables for readers who find this convenient. In the four classical calcite twin laws the mirror plane mapping the structures of the two intergrown individuals onto each other is also supposed to be the contact plane or interface plane between the twin domains. Since calcite is centrosymmetric, a mirror reflection twin law is symmetrically equivalent to a 180° rotation around the normal of the mirror plane. Thus, remarks of Bruno et al.^6^ who point out that the orientation relation for the twin with {012} contact plane is a 180° rotation around the {012} plane normal are correct, but are not at all in conflict with the earlier publications. According to their twin interface energy modeling, Bruno et al.^6^ conclude that the twin law of the {001} twin is 180°|<001> rather than m.{001}, but again, both orientation relationships are symmetry equivalent with respect to the point group of the crystal, as detailed explicitly by Hahn & Klapper^16^. In geologic and inorganic calcite the m.{001} twinning of calcite occurs as a growth twin only. The last three of the classical twinning modes can occur as growth twins^4^, but they are much more frequently generated due to plastic deformation by mechanical shear stresses (i.e. as deformation twins). Thus, they play a major role in assessing the deformation that a carbonate rock has undergone due to geological stresses or meteorite impact^7–9,17^. Schuster et al.^8^ and Seybold et al.^9^ define an “a-twin” of calcite generated by plastic deformation mechanisms. The “a-twin” is a “twin in a twin” as Richards^5^ put it when he argued that there are only the four twin laws in calcite, and all other reported laws are due to multiple twinning. Nevertheless, the “a-twin”^8,9^ is a regularly reoccurring orientation relationship with a defined contact plane, so it is fair to call it a twin law. A new, protein-induced growth twin in biomimetic calcite with the m.{108} twin law was reported by Pokroy et al.^18^, but their clear observation appears to have stayed a singular occurrence. The arguably best and only rigorous general definition of what is a crystallographic twin is that of Hahn & Klapper^16^ in Volume D of the International Tables of Crystallography. This definition is based purely on the structural-geometrical relation of the twins and is independent of the generation mechanism.

In recent electron backscatter diffraction (EBSD) investigations of the calcite which composes the tests of rotaliid foraminifera, Yin et al.^1^ and Lastam et al.^2,3^ found that the calcite produced by this important group of marine organisms shows intensive micro- to nanoscale twinning with an irregular dendritic, fractal-like twin-interface geometry. The twinned crystals themselves show serrate, dendritic fractal-like interfaces in the investigated cross-sections through the chamber walls^1–3^ which appears to be consistent with the “cogwheel structure” of calcite units which were found by Dijk et al.^19^ when imaging the outer surface of the chamber walls. The grain misorientation statistics, as obtained from the EBSD maps in our previous work^1–3^, showed a number of distinct peaks of misorientations at certain angles, the most prominent of which is a 60° rotation around the calcite c-axis ( = <001>). This operation is equivalent to 180°|<001> and m.{001} such that it corresponds to the above first-mentioned of the classical twin laws. However, the unique aspect of foraminiferal calcite is 180°|<001> *polysynthetic penetration twinning*, rather than the classical m.{001} contact twin observed in geological calcite. Apart from this classical calcite twin, there are additional maxima in the misorientation statistics^1–3^. For *Amphistegina lessonii* (d’Orbigny in Deshayes, 1830), Yin et al.^1^ obtained a prominent maximum in the misorientation angle statistics near the 77° misorientation around an axis close to [6 −6 1] according to the output of the HKL Channel5 EBSD software. This does not correspond to any known twin law of calcite and stimulated the work presented in the current paper. The intense calcite twinning of rotaliid foraminifera is a unique characteristic of this group of organisms. Biocalcite is usually untwinned. We are aware of only three previous reports of biocalcite twinning: Floquet & Vielzeuf^20^ observed a very systematic pattern of m.{104} nano-twinning in octocorals. The m.{018} twin law was proposed^21^ to exist in the so-called semi-nacre of the brachiopod *Novocrania anomala* (Müller, 1776) based on angles between the c-axis of adjacent crystals (rather than by a full 3-dimensional assessment of lattice orientation relationship), and m.{018} twinning was suggested^22^ between foliated and chalky calcite in the oyster *Magallana gigas* (Thunberg, 1793). In a recent paper by Castillo-Alvarez et al.^23^ twin lamellae are shown to exist in *Pinctada margaritifera* (Linnaeus, 1758) calcite, but again, their identification as m.{018} twins^23^ is based only on one misorientation angle and still requires a 3-dimensional orientation relationship analysis. In contrast to the scarce reports on biocalcite twinning, in biogenic aragonite micro- and nano-twinning has been known for almost a century since Bøggild’s (1930) pioneering work^24^. In biogenic aragonite twinning is a widespread feature^14,25–46^. When twinning occurs on the nanoscale as a systematic pattern in the hierarchical architecture of a shell, it contributes significantly to the toughness of the material^14^.

## Materials and methods

Foraminifera shells of the species *Amphistegina lesonii* were obtained from pebbles in the Gulf of Aqaba-Eilat, Red Sea and along the coast of the Pelagian islands, Central Mediterranian Sea. We investigated six specimens. Two were used for imaging shell surface structure and shell morphology, two specimens were microtome cut and the surfaces etched for imaging the internal structure of the shells and two specimens were microtome cut and microtome polished and the surfaces scanned with EBSD.

Electron backscattered diffraction (EBSD) data were obtained by embedding foraminiferal shells into EPON resin and cutting the embedded sample in the (as closely as possible) desired axial direction with a Leica Ultracut microtome. Subsequently, the cut was polished with the same ultramicrotome using a dry diamond knife (Diatome). The polishing step involves a series of sections with successively decreasing thickness at each step (90 nm, 70 nm, 40 nm, 20 nm, 10 nm). Each step was repeated 15 times. For the EBSD measurements or SEM imaging, the samples were coated with 4–6 nm of carbon and 6–8 nm of Pt/Pd, respectively. EBSD measurements and SE as well as BSE imaging were carried out with a Hitachi SU5000 FE-SEM, equipped with a Nordlys II EBSD detector. EBSD measurements were done with a step size of 180 nm and the data acquisition software AZTec (Oxford Instruments). Initial data evaluation to generate EBSD maps was performed with the CHANNEL 5 HKL software. For detailed statistical analysis the data of a single map of 1174 × 877 steps (1.029.598 individual data points) was exported with the CHANNEL 5 HKL software as a table containing the measurement position coordinates, Euler angles, band contrast, and mean angular deviation data of the Kikuchi pattern analysis. These data were further processed by PYTHON 3.0 codes written by ourselves. For SEM investigation of micro- and nano-scale internal structures, microtome-prepared flat surfaces as above were etched with a 0.1 M HEPES buffer (pH = 6.5) and 2.5% glutaraldehyde solutions for 90 and 120s, respectively. Etching was terminated by rinsing the samples three times in 100% isopropanol for 10s each. Subsequently, samples were critical-point dried and etched surfaces were coated with 4–6 nm of Pt/Pd. SE and BSE images were taken at 4 kV.

## Formalism applied to the analysis

**Fig. 1:**
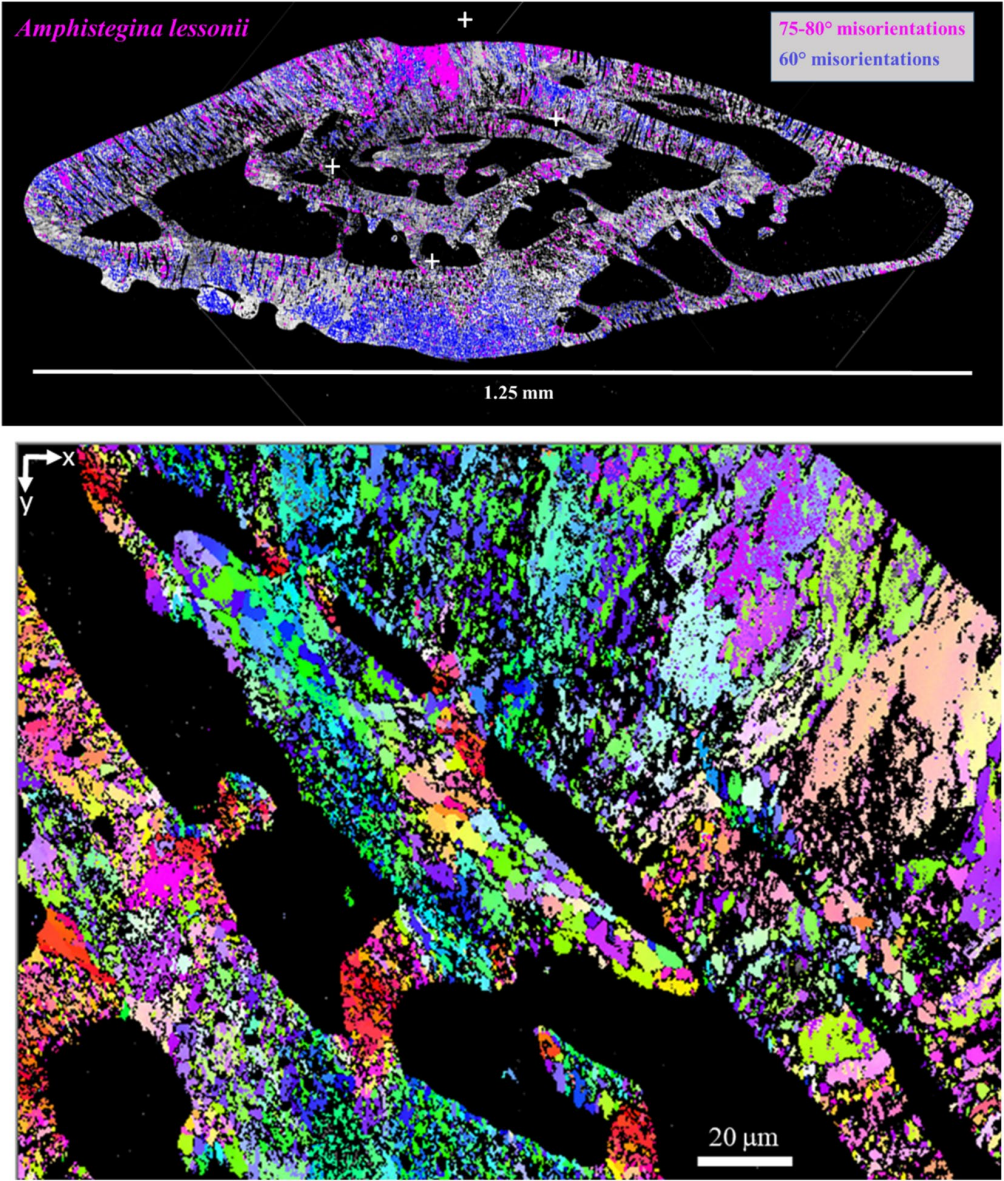
Top: Axial cross section of the shell of a specimen of *A. lessonii* (with dorsal side up); band contrast image) with indicated 60° twin boundaries and 75–80° misorientations as measured by EBSD^1^. The image is stitched together from 28 individual EBSD maps. Bottom: EBSD map investigated in detail in this paper with inverse pole figure coloring. The four white crosses in the top image indicate the location of the corners of the individual EBSD map shown in the bottom image. The axes of the used cartesian reference frame are indicated in the top left corner, the z-axis points away from the viewer.

EBSD measures crystal orientation in a ‘laboratory’ reference frame defined by the experimental set-up. We use a fixed reference frame which has the x-axis parallel to the horizontal axis of the SEM image and the equally oriented EBSD map. The y-axis is parallel to the vertical axis of the EBSD image (see Fig. 1, bottom). The z-axis is perpendicular to the sample surface, such that a right-handed coordinate system is formed with the origin at the top left corner of the EBSD scan. The orientation of the crystals within this frame can be described either by a triplet of Euler angles (φ_1_, ψ, φ_2_) or by an orthogonal 3 × 3 rotation matrix **Ω**. The components of **Ω** are related to the Euler angles by:

1 $$\begin{gathered} {\Omega _{{\text{11}}}}\,=\,{\text{cos}}{\varphi _{\text{1}}}{\text{ cos}}{\varphi _{\text{2}}} - {\text{sin}}{\varphi _{\text{1}}}{\text{cos}}\psi \;{\text{sin}}{\varphi _{\text{2}}} \hfill \\ {\Omega _{{\text{22}}}}={\text{ }} - {\text{sin}}{\varphi _{\text{1}}}{\text{ sin}}{\varphi _{\text{2}}}\,+\,{\text{cos}}{\varphi _{\text{1}}}{\text{cos}}\psi \;{\text{cos}}{\varphi _{\text{2}}} \hfill \\ {\Omega _{{\text{33}}}}\,=\,{\text{cos}}\psi \hfill \\ {\Omega _{{\text{12}}}}={\text{ }} - {\text{cos}}{\varphi _{\text{1}}}{\text{ sin}}{\varphi _{\text{2}}} - {\text{sin}}{\varphi _{\text{1}}}{\text{ cos}}\psi \;{\text{cos}}{\varphi _{\text{2}}} \hfill \\ {\Omega _{{\text{13}}}}\,=\,{\text{sin}}{\varphi _{\text{1}}}{\text{ sin}}\psi \hfill \\ {\Omega _{{\text{21}}}}\,=\,{\text{sin}}{\varphi _{\text{1}}}{\text{ co}}{\varphi _{\text{2}}}\,+\,{\text{cos}}{\varphi _{\text{1}}}{\text{ cos}}\psi \;{\text{sin}}{\varphi _{\text{2}}} \hfill \\ {\Omega _{{\text{23}}}}={\text{ }} - {\text{cos}}{\varphi _{\text{1}}}{\text{ sin}}\psi \hfill \\ {\Omega _{{\text{31}}}}\,=\,{\text{sin}}\psi \;{\text{sin}}{\varphi _{\text{2}}} \hfill \\ {\Omega _{{\text{32}}}}\,=\,{\text{sin}}\psi \;{\text{cos}}{\varphi _{\text{2}}} \hfill \\ \end{gathered}$$

The Euler angles rotate the crystal (as it is oriented in the sample in the laboratory reference frame) relative to the fixed standard orientation of the crystal lattice. To define this standard orientation, it is convenient to use the IEEE convention^47^, i.e. a lower diagonal matrix **L** converting the coordinates of the same vector from crystallographic fractional coordinates **x** onto Cartesian coordinates **y** in the form.

2 $${\mathbf{y}}\,=\,{\mathbf{Lx}}$$

with

3 $$\:\mathbf{L}=\:\left(\begin{array}{ccc}a\,\text{sin}\beta\:\:sin{\:\gamma\:}^{*}&\:0&\:0\\\:-a\,\text{sin}\beta\:\:cos\:{\gamma\:}^{*}&\:b\,\text{sin}\alpha\:\:&\:0\\\:a\,\text{cos}\,\beta\:&\:b\,\text{cos}\alpha\:\:&\:c\,\end{array}\right)$$

where a, b, c, α, β, γ are the lattice parameters, and γ* is the third angle of the corresponding reciprocal lattice a*, b*, c*, α*, β*, γ*. Accordingly, the basis vectors $\:\widehat{\mathbf{x}}$, $\:\widehat{\mathbf{y}}$, $\:\widehat{\mathbf{z}}$ of the Cartesian reference frame fixed in the crystal lattice with basis vectors (unit cell edges) **a**, **b**, **c** are related by the upper diagonal matrix

4 $${\mathbf{U}}\,=\,{{\mathbf{L}}^{ - \,{\text{1}}}}$$

as

5 $$(\hat {\mathbf{x}},\hat {\mathbf{y}},\hat {\mathbf{z}}){\text{ }}={\text{ }}\left( {{\mathbf{a}},{\mathbf{b}},{\mathbf{c}}} \right){\mathbf{U}}$$

Let the absolute orientation matrix of a crystal A be **Ω**_A_, then the atomic coordinates transformed from fractional coordinates **x** in standard orientation to the Cartesian reference space become

6 $$\:{\mathbf{y}}_{\text{A}}^{\:}=\left({\varvec{\Omega}}_{\text{A}}^{\:}{\mathbf{L}}_{\text{A}\:}^{\:}\right){\mathbf{x}}_{\:}^{\:}$$

The relative orientation, sometimes called misorientation, between two crystals or domains A and B with orientation **Ω**_A_ and **Ω**_B_, respectively, is thus defined by the misorientation matrix **R** as

7 $$\:{\varvec{\Omega}}_{\text{B}}^{\:}=\mathbf{R}{\varvec{\Omega}}_{\text{A}}\:\text{o}\text{r}\:\mathbf{R}={\:\varvec{\Omega}}_{\text{B}}^{\:}{\varvec{\Omega}}_{\text{A}}^{-1}$$

**R** is an orthogonal matrix with determinant + 1 for a rotation or − 1 for a rotoinversion (e.g. an inversion or a mirror-relationship). For a rotation, it is usually helpful to write the operation **R** as a rotation by an angle φ around a vector axis **v**:

8 $$\:\mathbf{R}==\left({\upphi\:}\left(\mathbf{R}\right)\:\right|\:\mathbf{v}\left(\mathbf{R}\right))$$

For the axis of a rotoinversion (or e.g. orientation of a mirror plane normal) **v** can be handled in the same way, after **R** is multiplied by the inversion matrix. Obviously, if **R** is the unit matrix or the inversion matrix, both $\:{\upphi\:}\left(\mathbf{R}\right)$ and $\:\mathbf{v}\left(\mathbf{R}\right)$ are not defined. If **v**$\:\left(\mathbf{R}\right)$ is normalized to a Cartesian unit vector with coordinates v_1_, v_2_, v_3_, the relation between matrix and angle/vector is

9 $$\begin{gathered} {{\text{R}}_{{\text{11}}}}\,=\,{{\text{v}}_{\text{1}}}{{\text{v}}_{\text{1}}}({\text{1}} - {\text{cos}}\varphi )\,+\,{\text{cos}}\varphi \hfill \\ {{\text{R}}_{{\text{12}}}}\,=\,{{\text{v}}_{\text{1}}}{{\text{v}}_{\text{2}}}({\text{1}}{\text{ }} - {\text{ cos}}\varphi ) - {{\text{v}}_{\text{3}}}{\text{ sin}}\varphi \hfill \\ {{\text{R}}_{{\text{13}}}}\,=\,{{\text{v}}_{\text{1}}}{{\text{v}}_{\text{3}}}({\text{1}}{\text{ }} - {\text{ cos}}\varphi )\,+\,{{\text{v}}_{\text{2}}}{\text{ sin}}\varphi \hfill \\ {{\text{R}}_{{\text{21}}}}\,=\,{{\text{v}}_{\text{2}}}{{\text{v}}_{\text{1}}}({\text{1}}{\text{ }} - {\text{ cos}}\varphi )\,+\,{{\text{v}}_{\text{3}}}{\text{ sin}}\varphi \hfill \\ {{\text{R}}_{{\text{22}}}}\,=\,{{\text{v}}_{\text{2}}}{{\text{v}}_{\text{2}}}({\text{1}}{\text{ }} - {\text{ cos}}\varphi )\,+\,{\text{cos}}\varphi \hfill \\ {{\text{R}}_{{\text{23}}}}\,=\,{{\text{v}}_{\text{2}}}{{\text{v}}_{\text{3}}}({\text{1}}{\text{ }} - {\text{ cos}}\varphi ) - {{\text{v}}_{\text{1}}}{\text{ sin}}\varphi \hfill \\ {{\text{R}}_{{\text{31}}}}\,=\,{{\text{v}}_{\text{3}}}{{\text{v}}_{\text{1}}}({\text{1}}{\text{ }} - {\text{ cos}}\varphi ) - {{\text{v}}_{\text{2}}}{\text{ sin}}\varphi \hfill \\ {{\text{R}}_{{\text{32}}}}\,=\,{{\text{v}}_{\text{3}}}{{\text{v}}_{\text{2}}}({\text{1}}{\text{ }} - {\text{ cos}}\varphi )\,+\,{{\text{v}}_{\text{1}}}{\text{ sin}}\varphi \hfill \\ {{\text{R}}_{{\text{33}}}}\,=\,{{\text{v}}_{\text{3}}}{{\text{v}}_{\text{3}}}({\text{1}}{\text{ }} - {\text{ cos}}\varphi )\,+\,{\text{cos}}\varphi \hfill \\ \end{gathered}$$

10 $$\:{\varphi}\left(\mathbf{R}\right)=\text{arccos}\left(\frac{Trace\left(\mathbf{R}\right)-1}{2}\right)\:\:\text{f}\text{o}\text{r}\,\text{det}\left(\mathbf{R}\right)=1$$

11 $$\:\mathbf{v}\left(\mathbf{R}\right)\:=\frac{1}{2sin{\varphi\:}}\left(\begin{array}{c}{R}_{32}-{R}_{23}\\\:{R}_{13}-{R}_{31}\\\:{R}_{21}-{R}_{12}\end{array}\right)\:\:\:\text{f}\text{o}\text{r}\:\text{d}\text{e}\text{t}\left(\mathbf{R}\right)=1$$

Equations (9), (10) and (11) can be applied to rotoinversions including mirror planes by appropriately multiplying **R** with the inversion matrix. Due to the symmetry of crystals, the expression of a relative orientation is not unique in terms of an **R**-matrix or $\:\left({\varphi\:}\right(\mathbf{R}),\mathbf{v}(\mathbf{R}\left)\right)$ quadruplet. To obtain at least a “symmetry-reduced” misorientation angle $\:{\varphi\:}$, the minimum $\:{{\varphi\:}}_{\text{m}\text{i}\text{n}}$ and its corresponding rotation vector $\:\mathbf{v}$ can be searched by a loop run over all symmetry-equivalent orientation relationships. Let (**S**, **t**)_i_ be the space group symmetry operators with their rotation or rotoinversion matrix **S**_i_ and translational part **t**_i_ such that a point or atom site at position **x**_1_ in fractional crystallographic coordinates is mapped onto

12 $$\:{\mathbf{x}}_{\text{i}}^{\:}={\mathbf{S}}_{\text{i}}^{\:}{\mathbf{x}}_{1}+\:{\mathbf{t}}_{\varvec{i}}$$

by the symmetry operation. With EBSD, the translational parts cannot be measured and only the $\:{\mathbf{S}}_{\text{i}}^{\:}$ matrices of the point group operations are relevant.

Expressing the symmetry operator $\:{\mathbf{S}}_{\text{i}}^{\:}\:$in the Cartesian sample reference frame (indicated by superscript “o”) for twin domain A we have

13a $$\:{\mathbf{S}}_{\text{A}\text{i}}^{\text{O}}={\left({\varvec{\Omega}}_{\text{A}}^{\:}{\mathbf{L}}_{\text{A}\:}^{\:}\right)\:\mathbf{S}}_{\text{i}}^{\:}\:{\left({\varvec{\Omega}}_{\text{A}}^{\:}{\mathbf{L}}_{\text{A}}^{\:}\right)}^{-1}\:$$

and likewise for twin domain B

13b $$\:{\mathbf{S}}_{\text{B}\text{i}}^{\text{O}}={\left({\varvec{\Omega}}_{\text{B}}^{\:}{\mathbf{L}}_{\mathbf{B}}^{\:}\right)\:\mathbf{S}}_{\text{i}}^{\:}\:{\left({\varvec{\Omega}}_{\text{B}}^{\:}{\mathbf{L}}_{\text{B}\:}^{\:}\right)}^{-1}\:$$

(Note that $\:{\mathbf{L}}_{\text{A}\:}^{\:}{\equiv\:\mathbf{L}}_{\text{B}\:}^{\:}$for crystals of the same phase).

Now there are two categories of symmetry-equivalent orientation relationships. For category I the application of a symmetry operator leaves the pole figures generated by the two crystals (or domains) A and B unchanged but relabels the individual peaks of the <uvw> or {hkl} by a symmetry-equivalent choice of the **a**, **b**, **c** basis vectors.

To find the minimum rotation angle and corresponding axis, it is sufficient for category I to search over all symmetry elements i = 1 to n_sym_ of the point group $\mathbb{P}$ of the crystal in one of the orientations (A or B), and the symmetry-equivalent misorientation matrices **R**_i_ are

14a $$\:{\mathbf{R}}_{\varvec{i}}\:=\:\mathbf{R}{\left({\mathbf{S}}_{\text{A}\text{i}}^{\text{O}}\right)}^{-1}\:\text{f}\text{o}\text{r}\:\text{a}\text{l}\text{l}\:{\mathbf{S}}_{\text{A}\text{i}}^{\:}\in\:\mathbb{\:}\mathbb{P}\left(\mathbf{A}\right)\:\text{w}\text{i}\text{t}\text{h}\:{\mathbf{S}}_{\text{A}\text{i}}^{\text{O}}\:\text{a}\text{s}\:\text{i}\text{n}\:\left(13\text{a}\right)\:$$

14b $$\:{\mathbf{R}}_{\varvec{i}}\:={\mathbf{S}}_{\text{B}\text{i}}^{\text{O}}\mathbf{R}\:\:\text{f}\text{o}\text{r}\:\text{a}\text{l}\text{l}\:{\mathbf{S}}_{\text{B}\text{i}}^{\:}\in\:\mathbb{\:}\mathbb{P}\left(\mathbf{B}\right)\:\text{w}\text{i}\text{t}\text{h}\:{\mathbf{S}}_{\text{B}\text{i}}^{\text{O}}\:\text{a}\text{s}\:\text{i}\text{n}\:\left(13\text{b}\right)$$

The rotation angle and axis are then given by the quadruplet $\:\left({{\varphi\:}}_{\:}\right({\mathbf{R}}_{\varvec{i}}),{\mathbf{v}}_{\:}({\mathbf{R}}_{\varvec{i}}\left)\right)$ as in Eqs. (10) and (11). Expressed in crystallographic fractional coordinates with respect to the lattice of the twin domains A and B, respectively, the rotation axis is

15 $$\:{\mathbf{v}}_{i}^{\text{A}}=\:{\left({\varvec{\Omega\:}}_{\text{A}}^{\:}{\mathbf{L}}_{\text{A}}^{\:}\right)}^{-1}{\:\mathbf{v}}_{\varvec{i}},\:{\mathbf{v}}_{i}^{\text{B}}=\:{\left({\varvec{\Omega\:}}_{\text{B}}^{\:}{\mathbf{L}}_{\text{B}}^{\:}\right)}^{-1}{\:\mathbf{v}}_{\varvec{i}}$$

For category II, an orientation relationship between crystals or domains A and B, **R**_AB_, is equivalent to an orientation relationship **R**_AC_ with crystal (domain) C differently oriented than B, if a symmetry element of A, which does not coincide with symmetry element of B and C in the given orientations ($\:{\varvec{\Omega\:}}_{\text{A},\text{B},\text{C}}^{\:}$), maps B and C onto each other. In category II, thus, in the pole figures of B and C the poles for the same {hkl} or <uvw>, respectively, have different positions. To find the equivalence, the search loop needs to run over the point group symmetry operators of both **A** and either **B** or **C**.

16 $$\:{\mathbf{R}}_{\text{A}\text{C}\text{i}\text{j}}\:={\mathbf{S}}_{\text{A}\text{i}}^{\text{O}}{\mathbf{S}}_{\text{C}\text{j}}^{\text{O}}{\mathbf{R}}_{\text{A}\text{C}}$$

The proof is given in the supplementary information. Axis and angle of rotation of **R**_ACij_ are calculated analogously to Eqs. (10) and (11).

## Results

### Overview

Figure 1 gives an overview of the location of twin walls in the whole shell cross-section of a specimen of the foraminifer *A. lessonii*, and it shows the EBSD map which we investigated in detail.

**Fig. 2:**
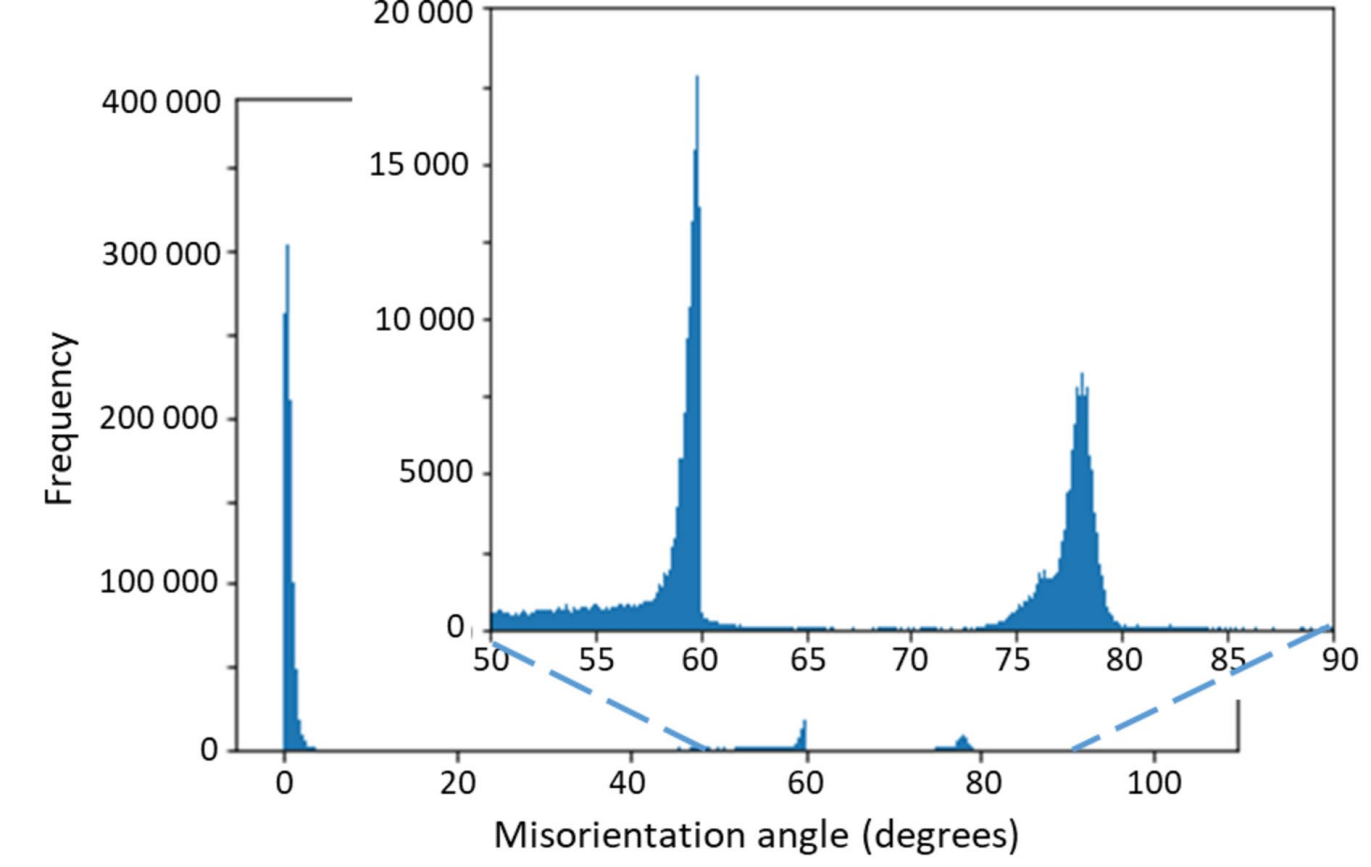
Histogram of misorientations in the map shown in the bottom image in Fig. 1. The peak near 0° is due to experimental reproducibility (ca. ± 0.2°) and mosaic spread of the crystals.

**Fig. 3:**
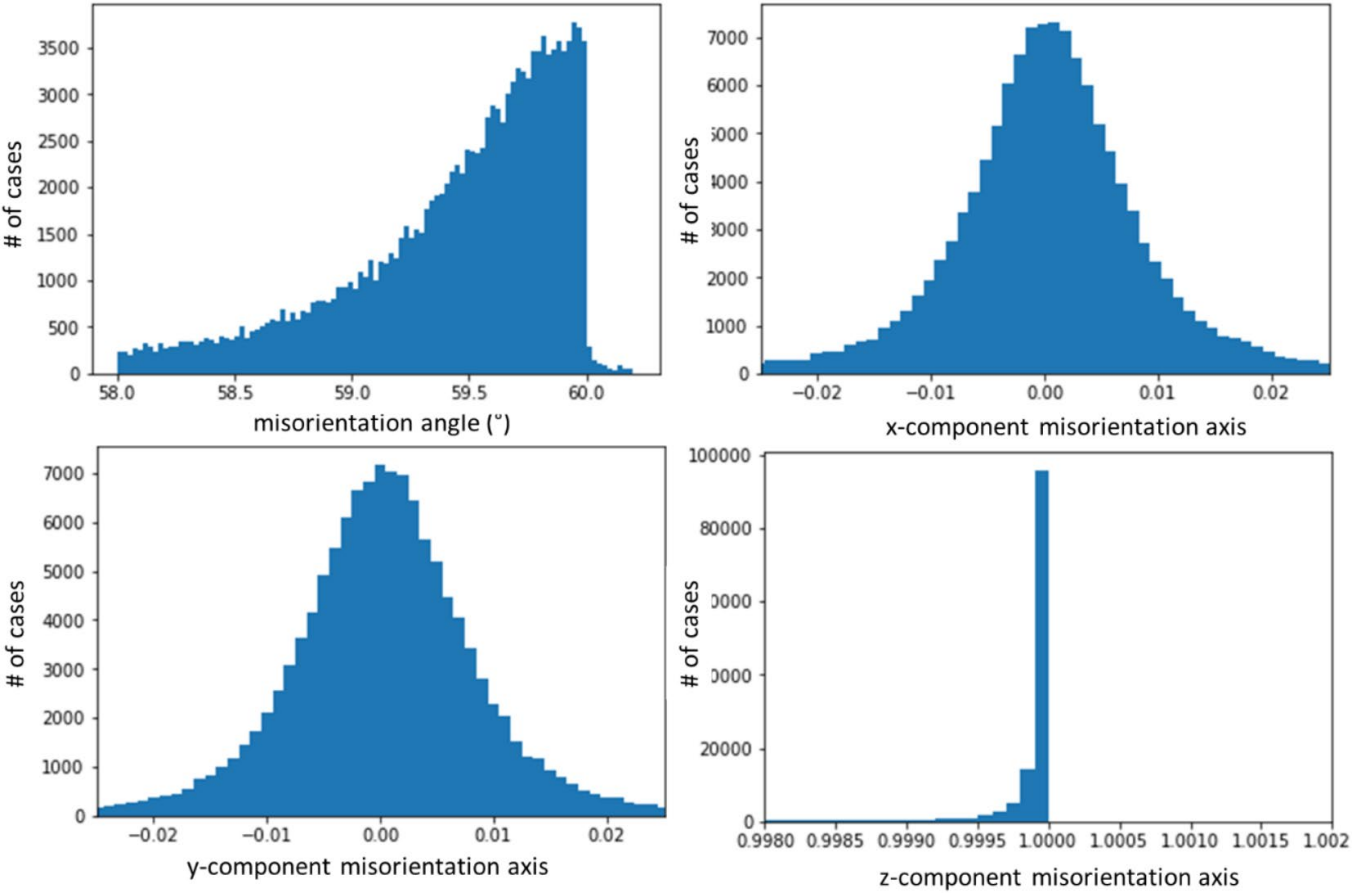
Misorientation axis and angle statistics for the 60° misorientation. The components of the misorientation axis represent a unit vector in the Cartesian reference frame fixed in the crystal lattice. Note that the sharp drop in the misorientation angle histogram is due to the trigonal symmetry of calcite.

Figure 2 displays the obtained histogram of misorientations. Except for the usual maximum at 0° misorientation, the histogram shows two maxima: one near 60° and one between 75° and 80° misorientation. In the latter angular range, there are a major peak at ca. 78° misorientation, and a smaller one at ca. 76° misorientation. The 60° peak appears skewed because the distribution shown in Figs. 2 and 3 is symmetry-reduced, i.e. from all symmetry-equivalent ($\:{\varphi\:}|\mathbf{v}$) expressions of misorientation, one of those with minimum $\:{\varphi\:}$ was chosen. The peak at 60° is due to the classical 60°|<001> twin misorientations. Due to the trigonal axis along <001>, a rotation of 60°+ε° around <001> can always be reduced to 60°- ε° around <0 0 −1>, where ε is a small positive number. Under the 60°|<001> peak there is a background of random misorientations, the frequency of which naturally increases with the circumference of the small circle described by the intersection of a cone with opening angle $\:{\varphi\:}$ and the unit sphere, i.e. proportional to sin^2^($\:{\varphi\:}$/2) until this function is cut off by the point group symmetry^48^. For the ${\bar3}2/\text{m}$ symmetry of calcite, the cut-off is very abrupt at 60°. For the peaks in the 75° to 80° range there is no abrupt cut-off in the present case, such that those peaks appear symmetric.

The different systematically reoccurring (twinning) orientation relationships were deconvoluted from the background of random misorientations and from each other in the following empirical way:

Step 1.) A filter window of ± 1° was set around each of the visually estimated maxima in the misorientation angle histogram. For the asymmetric distribution of the 60°|<001> twin, a filter window from 58.0° to 60.2° was set.

Step 2.) For the data falling into the selected windows of Step 1, the systematically reoccurring rotation axes contributing to this misorientation angle maximum were identified by generating the histogram for each of the three Cartesian components of the unit vector of the misorientation axis.

Step 3.) Finally, 4-condition window-filtering was applied for (i) the misorientation angle with a window width of 8° and (ii) for the three misorientation axis components with a window width of 0.05 for each Cartesian component of the unit vector of the rotation axis. These values for the width of the windows were deemed appropriate to select the correct contribution of each particular systematically reoccurring (angle | <axis>) misorientation in the misorientation angle histogram, i.e. to separate these from each other and from the background of random misorientations. We discuss the histograms for each case of the four-fold filtered data below.

### 60° twinning 60°|<001> equivalent to 180°|<001> and m.{001}

Figure 3 displays the histograms for the misorientation angle and the three Cartesian unit-vector coordinates of the misorientation axes related to the misorientations in the range between 58° and 60.2°. The misorientation axis obtained by the filtering procedure for this orientation relationship is clearly <001> (see Table 1 for experimental standard deviations).

**Table 1:** Misorientation angles ω_mis_ and corresponding misorientation axis components in fractional crystallographic coordinates [*uvw*] as well as corresponding mirror plane (*hkl*) for the systematically reoccurring orientation relationships or twin relationships. For the integer coordinates, the equivalent bravais notations are also given. The standard deviations of the [uvw] values are given in brackets, with respect to the last digit of the [*uvw*] value. The [*uvw*] values in square brackets give the standard deviation of coordinates converted to an angle. The table also lists the closest operation with integer crystallographic indices and a 2-fold axis or mirror operation, respectively.

ω_mis_ (°)		u	v	w	
60.0(5)		0.00(4) [1.7°]	0.00(5) [1.6°]	1.000(4) [0.0°]	in crystals A and B
	0° from	0	0	1	
76.6(8)		6.0(1) [0.4°]	− 6.0(1) [0.3°]	0.99(2) [0.7°]	
	0.3° from	6	– 6	1	in crystal A
	0.3° from	6	− 6	1	in crystal B
	Bravais	[6 −6 0 1]			
The above approximately equivalent to the two following operations:					
180° around	2.3° from	4	5	1	in crystal A
	Bravais	[1 2 −3 1]			
	2.3° from	5	4	− 1	in crystal B
	Bravais	[2 1 −3 1]			
mirror plane	5.1° from	(1 2 9)			in crystal A
	Bravais	(1 2 −3 9)			
		(2 1 −9)			in crystal B
	Bravais	(2 1 −3 9)			
78.2(6)		8.92(5) [0.2°]	8.92(8) [0.4°]	1.02(3) [0.7°]	
	0.5° from	9	9	1	in crystal A
	0.5° from	9	9	1	in crystal B
	Bravais	[3 3 −6 1]			

**Fig. 4:**
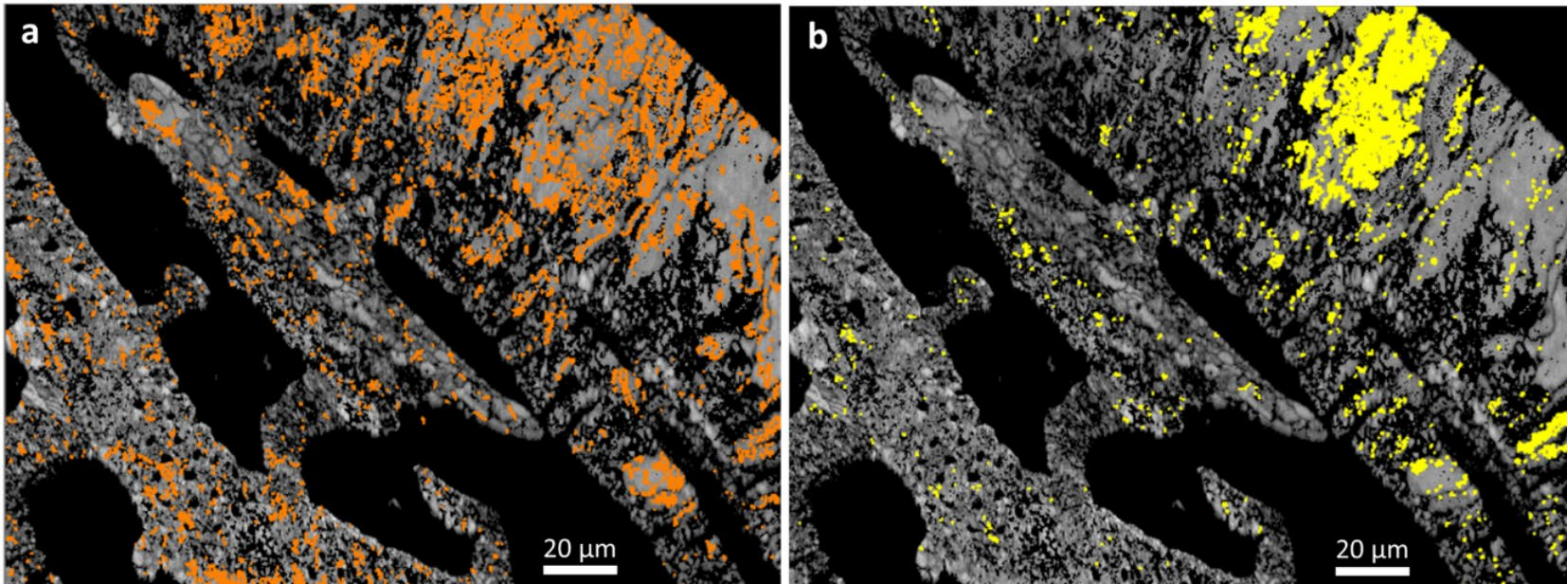
Spatial distribution of the systematically reoccurring misorientations in the investigated map. (**a**) 60° twin boundaries, (**b**) misorientations in the 75–80° interval.

Figure 4a shows the spatial distribution of the observed 60°|<001> twinning in the selected scan area.

**Fig. 5:**
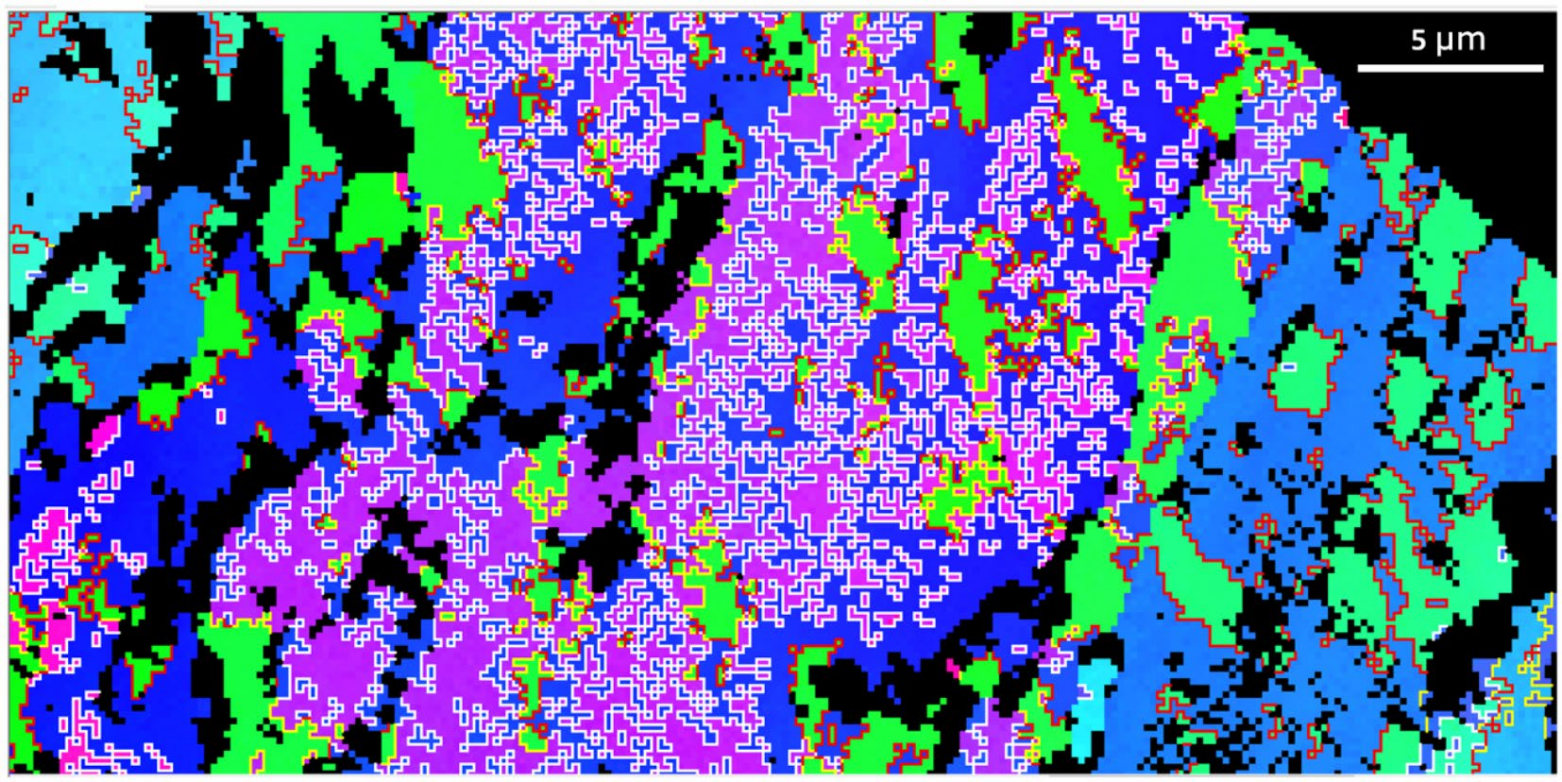
Spatial distribution of the systematically reoccurring misorientations in a part of the investigated map where their concentration is highest. Red lines: 60°|<001> twin boundaries, yellow lines: 76.6°|<6 −6 1>, white lines: 87.2°|<991> misorientations. The colors in the background are an inverse pole figure coloring scheme (z-orientation) for crystal orientation. The rectangular pattern arises from the 180 nm steps (pixels) in which the EBSD map was collected. Note that the “yellow” walls occur between domains for which one is related to a third domain by a “red” wall, and the other to the third domain by a “white” wall.

A magnification of this part of the map is shown in Fig. 5. The 60°|<001> twin domains are in the size range of 1 to 5 micrometers and they constitute a polysynthetic penetration twin rather than “large” twin domains on the scale of the extent of the twinned crystal entity (10–50 micrometers). Note that neither the 60°|<001> twin walls nor the 76.6°|<6 −6 1> and 87.2°|<991> interfaces follow regular “rational” crystallographic planes; all twin interfaces have a dendritic fractal-like appearance.

### Analysis of the peaks in the 75–80° misorientation interval

**Fig. 6:**
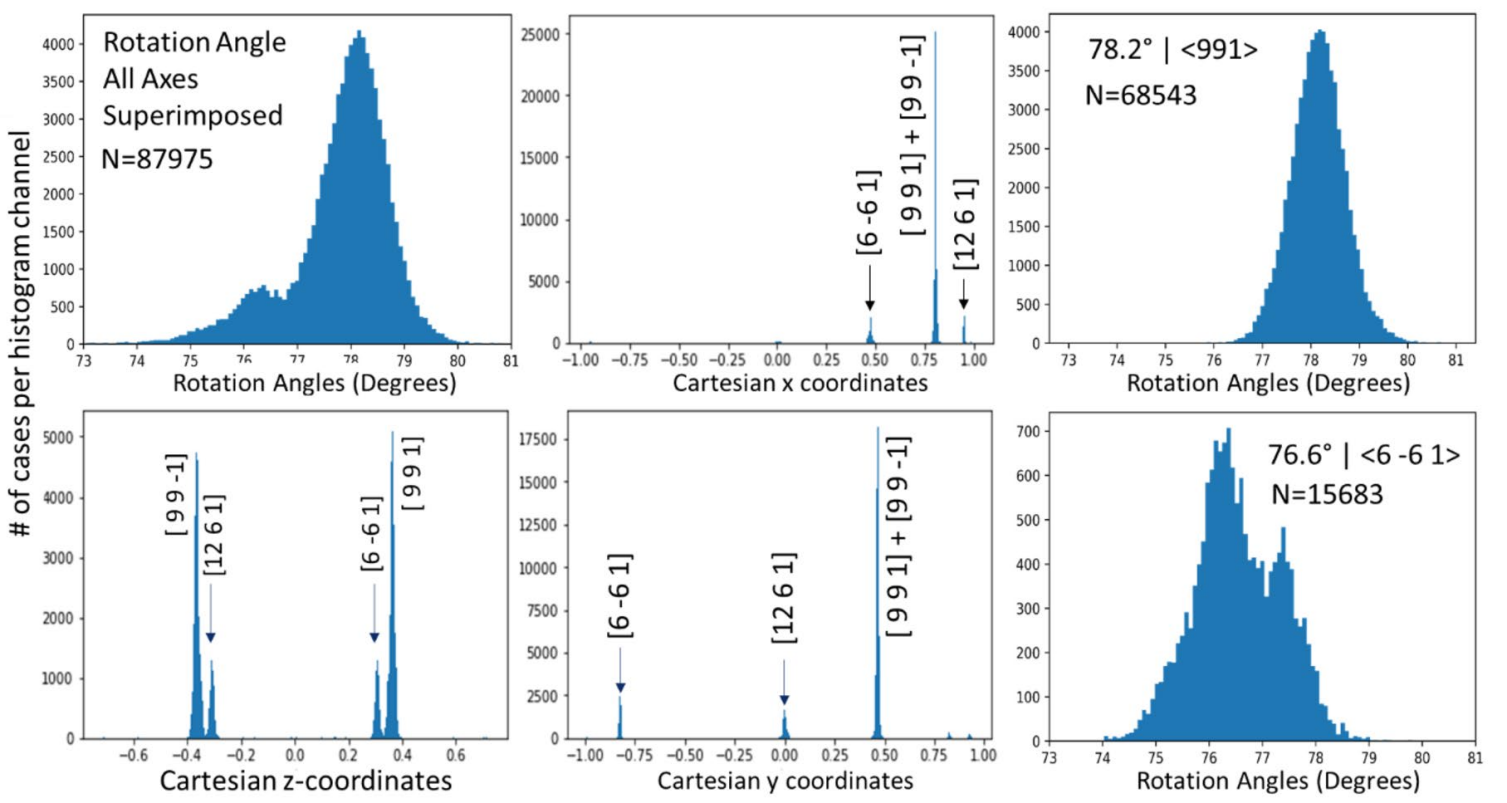
Statistics of the misorientation angle and axis components (x, y, z in Cartesian space fixed in the crystal as defined by Eq. 2) in the 73°–81° interval of misorientations. The corresponding crystallographic rational indices for each of the axes are given above the observed peaks in the data. The rotation angle distribution decomposed into the 78.2°|<991> and the 76.6° | <6 −6 1> components is shown on the right. The total number of data points N in each rotation angle histogram is also indicated in the figure. In all cases, the vertical axis is the number of cases per histogram channel.

**Fig. 7:**
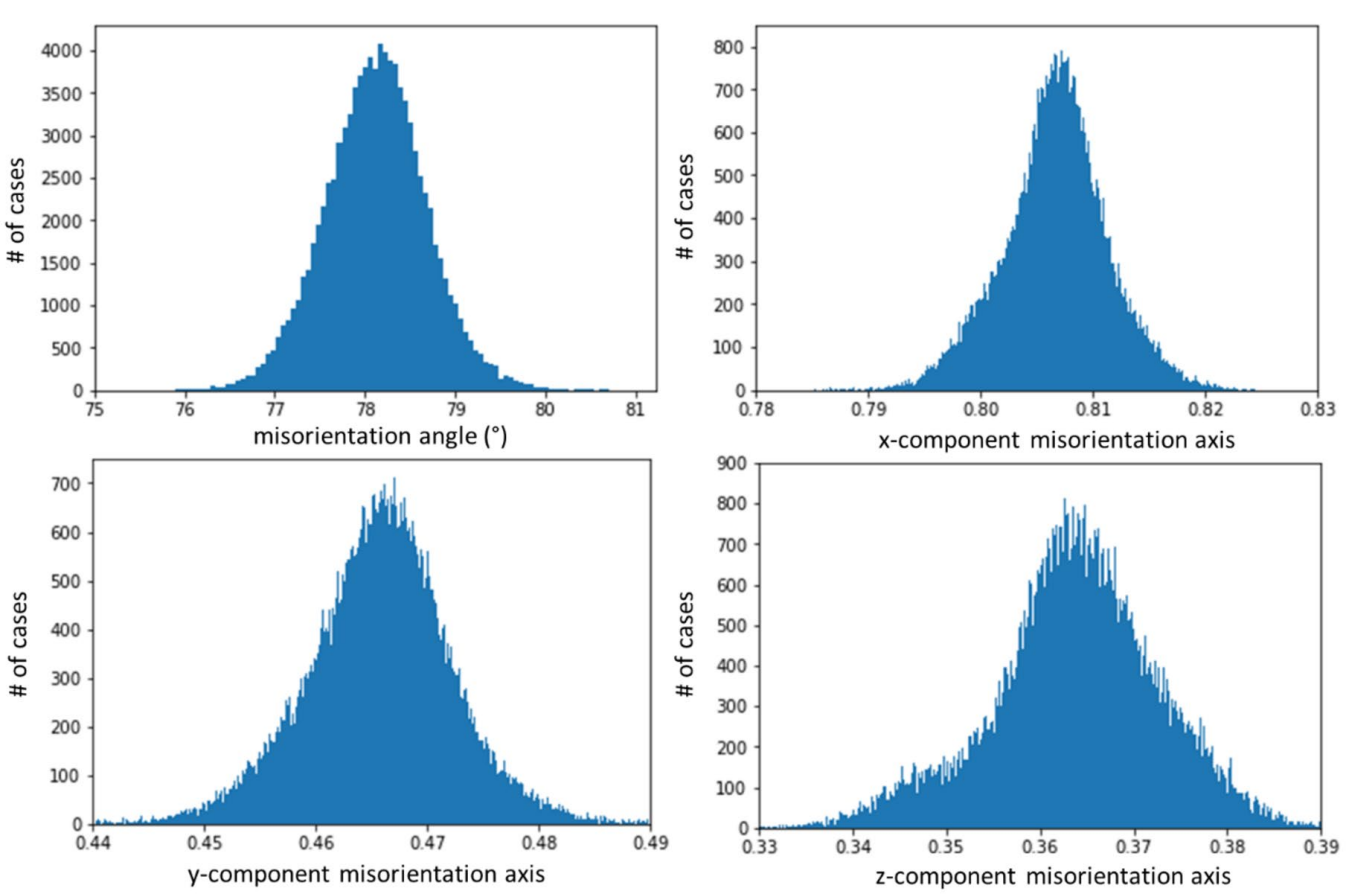
Misorientation axis and angle statistics for the 78.2° misorientation. The displayed components of the misorientation axis represent a unit-vector in Cartesian sample space. For the z component, the negative and positive z-values are displayed as |z|.

**Fig. 8:**
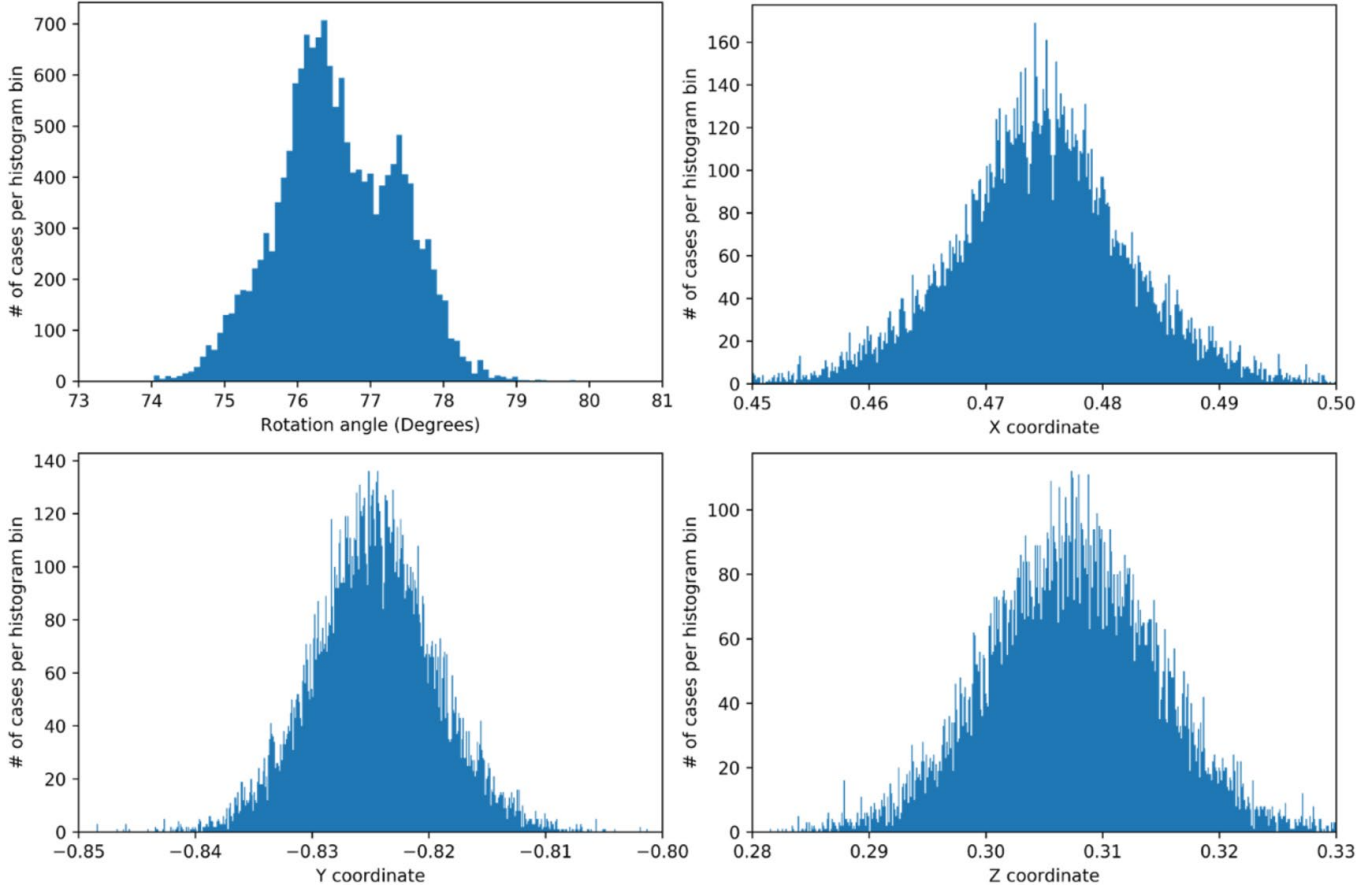
Misorientation axis and angle statistics for the 76.6° | <6 −6 1> misorientation. The displayed statistics for the vector components of the misorientation axis are expressed as unit-vector in Cartesian (Eq. 2) sample space. All <6 −6 1> symmetry-equivalent contributions have been merged by symmetry-transformation onto [6 −6 1].

The spatial distribution pattern of the 75–80° misorientations is shown in Figs. 4b and 5. The domain size for this pattern of alternating crystal lattice orientations is between the 180 nm pixel size of the measurement and ca. 2 μm. For the misorientation angular range between 75° and 80°, four misorientation axes were contributing (Fig. 6): [9 9 1], [9 9 −1], [6 −6 1], and [12 6 −1], in crystallographic coordinates referring to the hexagonal setting of the unit cell. The two former and the two latter twin orientations are symmetry-equivalent by category II. The contributions of these axes overlapping in the 75°–80° range were separated by the filtering procedure described in Sect. 4.1 of this paper. The filtering procedure placed the mean value of the major peak at 78.1(6)°|<991> (Figs. 6 and 7) and the minor peak at 76.6(8)°, <6 −6 1> (Figs. 6 and 8). The limits of uncertainty obtained for the present experiment and interpretation are given in Table 1 both (i) as experimental standard deviations and (ii) as angular deviations from rational crystallographic directions or plane normal.

For the 76.6°|<6 –6 1> relationship, the misorientation angle distribution is bimodal (Figs. 6 and 8). The origin of the bimodality could not be solved so far. We did not find a correlation between the bimodality to coordinates of the rotation axes or to the position in the map.

With a 2.3° deviation (three standard deviations), the 76.6°|<6 –6 1> misorientation is symmetry-equivalent to a 180° rotation around the rational <451> axis. With a 5.1° deviation (7 standard deviations), it is symmetry-equivalent to a rational mirror operation on a {129} plane.

For the 78.2|<991> orientation relation, the symmetry analysis did not produce any symmetry-equivalent rotations with “crystallographic” rotation angles (60°, 90°, 120°, 180°) nor with a mirror plane.

**Fig. 9:**
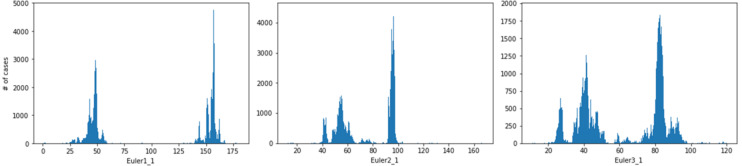
Euler angles associated with the 78.2° | <991> misorientation relationship or non-classical twinning. The distribution shows that the EBSD detection of this misorientation is not limited to a specific absolute crystal orientation.

**Fig. 10:**
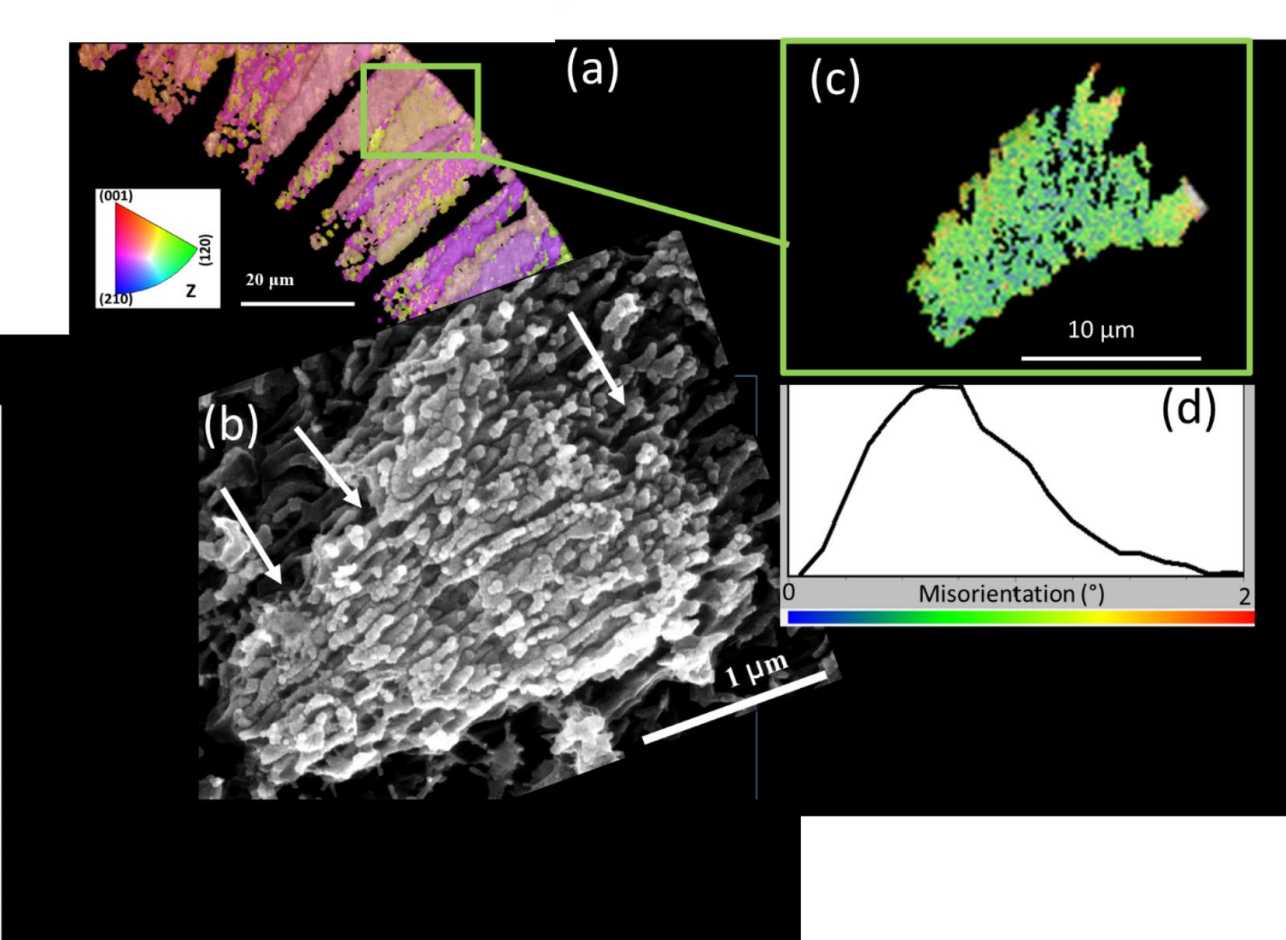
Nanofibrous nature and crystallographic co-orientation of foraminiferal calcite. (**a**) EBSD map, (**b**) selectively etched surface showing a sheaf of nanofibrils, (**c**, **d**) misorientation plot of a randomly selected sheaf-like crystal showing the internal crystallographic co-orientation of the fibrils with a mosaic spread of ~ 1° FWHM. The white arrows in (**a**) indicate growth stages where the crystal grows through sheets of organic matrix.

## Discussion of the observed twin- or orientation relationships

### Comparison with classical calcite twin laws

An overview of the geometry of the known classical twin laws is given in Table 2 together with the new non-classical systematically reoccurring orientation relationships found in *A. lessonii* analyzed in the present paper. Note that a twin law cannot be identified by only one misorientation angle but it rather needs specification of the rotation angle and the rotation axis (or the rotation matrix or three Euler angles of misorientation).

**Table 2:** Classical^4–9,18,]^ and non-classical new calcite twins and their smallest EBSD misorientation angle Φ and corresponding axis. Refer to the supplementary material for a graphical illustration. Acronym sor = *s*ystematically reoccurring *o*rientation *r*elationship. The so-called a-type twins are “twins in twins”, the two sequential classical twin operations are indicated.

Twin Name	Notation	Twin mirror plane or coincidence site lattice plane (CSLP)	180° Twin axis (pole to twin plane)	EBSD misorientation angle φ and misorientation axis	
{001} contact twin or penetration twin	Vector	{001}	<001>	60°	<001>
	Bravais	{0001}	<0001>		<0001>
e-twin	Vector	(018)	0.6° from [121]	78.1°	0.7 ° from [4 2 1]
	Bravais	(0 1 −1 8)	0.6° from [0 1 −1 1]		0.7 ° from [2 0 −2 1]
r-twin	Vector	(104)	0.7° from [421]	103.9°	0.6 ° from [4 −4 1]
	Bravais	(1 0 −1 4)	0.7° from [2 0 2 1]		0.6 ° from [4 −4 0 1]
f-twin	Vector	(012)	0.6 ° from [4 8 1]	78.8°	[2 −2 1]
	Bravais	(0 1 −1 2)	0.6° from [0 4 −4 1]		[2 –2 0 1]
a-type r$\otimes$e	Vector	first e then r		38.2°	[1 0 0]
	Bravais				[2 –1 −1 0]
a-type f$\otimes$r	Vector	first r then f		35.5°	[100]
	Bravais				[2 −1 −1 0]
{108} Pokroy	Vector	(108)	0.6 ° from [2 1 1]	78.1°	0.7 ° from [2 −2 1]
	Bravais	(1 0 −1 8)	0.6° from [ 1 0 –1 1]		0.7 ° from [2 –2 0 1 ]
	Vector		0° from [8 4 –1]		
	Bravais		0° from [4 0 −4 −1]		
sor <6–6 1>	Vector	5.1° from (129)	2.3° from [4 5 1]	76.6	[6 −6 1]
	Bravais	5.1° from (1 2 −3 9)	2.3° from [1 2 −3 1]		[6 −6 0 1]
sor <9 9 1>	Vector	CSLP (−7 5 18)	–	78.2	[9 9 1]
	Bravais	CSLP (−7 5 2 18)	–		[3 3 −6 1]

### The classical 60°|<001> twinning in *Amphistegina Lessonii*

The 60°|<001> (= m.{001}) twinning is common as a contact twin in inorganic calcite^4,5^. However, in the case of foraminiferal calcite, the 60°|<001> twinning has a polysynthetic constitution with interpenetrating micro- and submicro-domains. Hahn & Klapper^16^ state that “for calcite only {0001} contact twins are found” whereas “for isotypic FeBO_3_ … this twin law always, without exception, forms penetration twins.” Thus, the observed polysynthetic penetration twinning is a new and very prominent feature of foraminiferal calcite and has been observed for most species of foraminifera examined so far^1–3^. The calcite of planktonic rotaliid foraminifera shows the highest abundance of 60°|<001> twin walls^2^; in benthic foraminifera, twins are much less abundant, and the more spherical the shape of the species of foraminifera, the higher the spatial density of these twins. For the 60°|<001> twin law, the twin interface wall can take any orientation - similar to an inversion twin or an antiphase domain boundary^49^, as any (hkl) interface plane will give a high density of common lattice points of the two twin domains.

The intensive twinning correlates with a nanofibrillar internal constitution of the calcite crystal units that have been described by Lastam et al.^2,3^ (Fig. 10). Within the sheaves of calcite nanofibrils visible in Fig. 10, the nanofibrils are fully co-oriented crystallographically in 3 dimensions, with the calcite c-axis along the morphological axis of the nanofibrils^2,3^. From sheaf to sheaf, the a-b-axes orientation changes randomly to produce an axial texture (i.e. random distribution of crystallite orientation around <001> ) in the shell. The 60°|<001> twinning occurs between bundles of nanofibrils. Therefore, calcite nucleation is - at least to some part - directed by the organic matrix of the primary organic sheet in which the morphology of crystallographically co-oriented nanofibrils develops. This control mechanism appears to be indifferent with respect to the two orientation variants related by the 60°|<001> twin law.

### The 76.6° <6 −6 1> misorientation in *Amphistegina Lessonii*

This misorientation is equivalent within 2.3° (a few standard deviations) to a 180° rotation around the rational <451> axis, and, with a 5.1° deviation (7 standard deviations), it is symmetry equivalent to a rational mirror operation on a {129} plane. Both deviations are well within the typical misorientation range of small-angle boundaries. For small-angle boundaries, there are coherent rational interfaces over small spatial regions, and these coherent regions are separated by dislocations which are arrayed periodically. Small-angle grain boundaries are a typical growth feature of biological calcite and aragonite, and they are particularly frequent if Mg^2+^ is present in the solution from which calcite grows^50,51^.

### The 78.2° | <9 9 1> misorientation in *Amphistegina Lessonii*

The 78.2°|<9 9 1> misorientation is a systematically reoccurring orientation relationship in the calcite in the shell of the foraminifera *A. lessonii*. It does not just occur in a singular calcite grain, but exists in twinned crystals with a range of different absolute orientations (i.e. Euler angles, Fig. 9).

Like the 76.6°|<6 −6 1> misorientation, it does not correspond to any of the known classical twinning patterns of calcite (Table 2). Further, the direction [9 9 1] is 20.8° from [110] and thus sufficiently far away from this (or other) simple rational directions, and there is no “classical crystallographic” operation (60°, 90°, 120°, 180°, or m) that is symmetry-equivalent to the (78.2° | <9 9 1> ) rotation. In spite of the high indices of the rotation axis, there is a common crystallographic plane of the two twin partners, in which lattice points are approximately coincident within less than 0.1Å. For 78.2°|[991], this is the (−7 5 18) plane for crystal A, and the (−5 7 −18) plane for crystal B. Coincident calcium sites in this common plane are listed in Table 3. As the hexagonal setting relates to an R-centered unit cell, some of the sites have fractional (non-integer) coordinates. Due to this, we also list the Ca-coordinates with respect to the primitive rhombohedral cell, which have integer values throughout. The shortest coincident vectors are [−3, 0, 0]_rhomb_ and [2, −1, 0]_rhomb_ with a distance of 12.14 Å from the origin point for the lattice parameters used in this work (see Introduction). This coincidence site lattice is not particularly dense, but perhaps sufficiently dense to call this relationship a twin relationship.

**Table 3:** Common lattice vectors of crystal A and B in relative orientation 78.2°|[991]. Coordinates *uvw* are given, with respect to both the primitive rhombohedral unit cell and the hexagonal R-centered unit cell. Dist: distance between the lattice point in crystal A to the corresponding lattice point in crystal B. D: distance from the origin. Strain: Dist/D in per Mille. The shortest common lattice vectors are highlighted in bold.

		Rhombohedral coordinates			Rhombohedral coordinates			Hexagonal coordinates			Hexagonal coordinates			
		Crystal A			Crystal B			Crystal A			Crystal B			
Dist[Å]	Strain	u	v	w	u	v	w	u	v	w	u	v	w	D[Å]
0.000	0.00	− 10	− 1	8	− 10	− 1	8	− 9.0000	− 9.0000	− 1.0000	− 9.0000	− 9.0000	− 1.0000	48.03
0.000	0.00	10	1	− 8	10	1	− 8	9.0000	9.0000	1.0000	9.0000	9.0000	1.0000	48.03
0.048	0.99	− 10	2	2	− 6	− 6	6	− 8.0000	− 4.0000	− 2.0000	− 4.0000	− 8.0000	− 2.0000	48.57
0.012	0.31	− 8	− 2	7	− 9	0	6	− 7.1667	− 8.3333	− 0.8333	− 8.1667	− 7.3333	− 0.8333	38.62
0.036	0.99	− 8	1	1	− 5	− 5	4	− 6.1667	− 3.3333	− 1.8333	− 3.1667	− 6.3333	− 1.8333	36.43
0.024	0.77	− 5	− 2	7	− 7	2	5	− 5.0000	− 7.0000	0.0000	− 7.0000	− 5.0000	0.0000	31.16
0.024	0.99	− 5	1	1	− 3	− 3	3	− 4.0000	− 2.0000	− 1.0000	− 2.0000	− 4.0000	− 1.0000	24.29
0.072	1.68	− 5	4	− 5	1	− 8	1	− 3.0000	3.0000	− 2.0000	3.0000	− 3.0000	− 2.0000	42.85
0.036	1.32	− 3	− 3	6	− 6	3	3	− 3.1667	− 6.3333	0.1667	− 6.1667	− 3.3333	0.1667	27.29
**0.012**	**0.99**	**− 3**	**0**	**0**	**− 2**	**− 2**	**1**	**− 2.1667**	**− 1.3333**	**− 0.8333**	**− 1.1667**	**− 2.3333**	**− 0.8333**	**12.14**
0.060	1.75	− 3	3	− 5	2	− 7	0	− 1.1667	3.6667	− 1.8333	3.8333	− 1.3333	− 1.8333	34.32
0.048	1.69	0	− 3	6	− 4	5	2	− 1.0000	− 5.0000	1.0000	− 5.0000	− 1.0000	1.0000	28.53
0.048	1.69	0	3	− 6	4	− 5	− 2	1.0000	5.0000	− 1.0000	5.0000	1.0000	− 1.0000	28.53
0.060	1.75	2	− 4	5	− 3	6	0	0.8333	− 4.3333	1.1667	− 4.1667	0.6667	1.1667	34.32
**0.012**	**0.99**	**2**	**− 1**	**0**	**1**	**1**	**− 1**	**1.8333**	**0.6667**	**0.1667**	**0.8333**	**1.6667**	**0.1667**	**12.14**
0.036	1.32	2	2	− 6	5	− 4	− 3	2.8333	5.6667	− 0.8333	5.8333	2.6667	− 0.8333	27.29
0.072	1.68	5	− 4	5	− 1	8	− 1	3.0000	− 3.0000	2.0000	− 3.0000	3.0000	2.0000	42.85
0.024	0.99	5	− 1	− 1	3	3	− 3	4.0000	2.0000	1.0000	2.0000	4.0000	1.0000	24.29
0.024	0.77	5	2	− 7	7	− 2	− 5	5.0000	7.0000	− 0.0000	7.0000	5.0000	− 0.0000	31.16
0.036	0.99	7	− 2	− 1	4	4	− 4	5.8333	2.6667	1.1667	2.8333	5.6667	1.1667	36.43
0.012	0.31	7	1	− 7	8	− 1	− 6	6.8333	7.6667	0.1667	7.8333	6.6667	0.1667	38.62
0.048	0.99	10	− 2	− 2	6	6	− 6	8.0000	4.0000	2.0000	4.0000	8.0000	2.0000	48.57
0.000	0.00	− 10	− 1	8	− 10	− 1	8	− 9.0000	− 9.0000	− 1.0000	− 9.0000	− 9.0000	− 1.0000	48.03

		Common plane in rhombohedral setting						Common plane in hex setting						
		Crystal A			Crystal B			Crystal A			crystal B			
		h	k	l	h	k	l	h	k	l	h	k	l	
		3	10	5	− 7	− 2	− 9	− 7	5	18	− 5	7	− 18	

## Conclusions

**(1) The newly described systematic orientation relationships 76.6°|<6 −6 1> and 78.2° | <9 9 1> can be regarded as twins**

The 76.6°|<6 −6 1> relationship is sufficiently close to classical 180° or m operations, respectively, albeit on axes or planes that have not been previously described for twins in calcite, i.e. (180°|<451> and m.{129}).

The new 78.2° | <9 9 1> relationship, however, is more out of the ordinary. Janovec, Hahn, Klapper and Přívratská^16,49,52^ give an extensive review of twinning and domain structures in Volume D of the International Tables of Crystallography. They highlight numerous examples, give a critical assessments of phenomena related to the subject, and define crystal twinning as follows (Chap. 3.3.2.1 of Hahn & Klapper(2013)^16^):

*An intergrowth of two or more macroscopic*,* congruent or enantiomorphic*,* individuals of the same crystal species is called a twin*,* if the orientation relations between the individuals occur frequently and are ‘crystallographic’. ….*

The authors define their term *‘crystallographic’* as follows:

*The orientation relationship is defined as crystallographic … if the following two minimal conditions are simultaneously observed*:

*(i) at least one lattice row (crystal edge) [uvw] is ‘common’ to both partners …*.

*(ii) at least two lattice planes (crystal faces) (hkl)*,* one from each partner*,* are ‘parallel’*,* but not necessarily ‘common’.*.

(The quotation marks used here are in the original text^16^. Hahn & Klapper^16^ define their term ‘*common*’ as:

… *all lattice points in the common row or common plane*,* respectively*,* are coincident or can be made coincident by a shift of origin*.

(refer to Hahn & Klapper^16^ for the complete text.)

The 78.2°|<9 9 1> orientation relationship of calcite certainly matches the definition of Hahn & Klapper^16^ as there are both a common lattice row and common lattice planes (see Tables 2 and 3). The Hahn & Klapper^16^ definition may appear clumsy. However, it is necessarily so to account for the diversity of reoccurring “regular” intergrowths of crystals of the same phase, which have been described and accepted as twins over the past two centuries of crystallography and mineralogy.

**(2) The classical and non-classical twins observed in** ***Amphistegina lessonii*** **are growth twins mediated by the specific organic matrix in which they evolve**

Twins originating from crystallographic symmetry changes occurring at structural phase transitions (transformation twins) or from shear deformation by coordinated motion of dislocations during plastic deformation (mechanical twins) are well studied and well understood by stringent theoretical concepts (see^49,52^ for an excellent and comprehensive review). Effects occurring during crystal nucleation and growth that lead to twinning (growth twins), however, are much more random and elusive in nature. They are subject both to growth defects evolving in the crystal itself, and to mechanisms occurring at the interface with the substance on which a heterogeneous nucleation of the crystallizing phase occurs. For such mechanisms, spurious amounts of certain components may be sufficient to steer the growth process in one or other direction. The in-vitro growth experiment of Pokroy et al.^18^ showed without doubt that a protein can cause growth twinning in calcite with an orientation relationship that is non-classical with respect to the calcite literature. For the present case of rotaliid foraminifera there are three main observations which make the control of calcite nucleation and growth by the organic matrix of the chamber wall evident:

(i) The three types of growth twins which are described in the present paper, i.e. extensive formation of 60°|<001> *polysynthetic penetration* twins, as well as the crystallographically irrational but reoccurring 76.6°|<6 −6 1> and 78.2°|<9 9 1> orientation relationships have not been observed at all in geologic or synthetic calcite and they are also essentially absent in the calcite of other well-investigated marine organisms such as brachiopods (see Simonet Roda et al.^53^ and references therein), molluscs (see Checa^54^, and references therein), echinoderms^55–57^, and coccolithophores^58^, with corals as a rare proven exception^20^. Likewise, calcite grown from solution essentially does not show these features (e.g^59–64^ and references therein).
(ii) The axial preferred crystallographic orientation (texture) of foraminiferal calcite is already present right near the nucleation sites on the primary organic sheet (POS) (Fig. 11 of Lastam et al.^2^). This texture is therefore not created by growth selection, but it is generated by oriented nucleation on the organic template.
(iii) The calcite crystals of rotaliid foraminifera have a distinct nanofibrillar internal structure (Fig. 10 of the present paper and^1–3^), which is not found for the calcite formed by other marine organisms or inorganic calcite (e.g^53–58^ and references therein). Calcite growing though the porous networks of gel-like organic matrices easily incorporates the organic substance^59–64^. Thus, the nanofibrillar internal structure of foraminiferal calcite indicates crystal growth in an organic matrix that provides linear nanoscale channels elongated in the direction perpendicular to the POS.

Finally, with the assumption that the molecular-scale mechanism of the oriented nucleation on the organic substrate is not selective between the twin-related orientations, which would obviously provide very similar interfaces with the organic matrix, there is an obvious and simple explanation for the extensive twinning of foraminiferal calcite.

Nagai et al.^65^ and Tyszka et al.^66^ showed that the foraminiferal calcite grows between the outer layers of an organic triple-membrane. The POS, on which the nucleation occurs, is sandwiched between the inner and outer lamellopodium. Both lamellopodia are cytoplasmic and contain actin-meshworks^66^. For foraminifera the actin in or on the initially purely organic chamber wall is a prominent feature which is decisively important for the entire chamber morphogenesis of foraminifera^65,66^. We suspect that the unique fibrillar microstructure and polysynthetic twinning might be related to the organization of organic matrices controlled by the actin network which is associated with transmembrane and other related proteins. The foraminiferal biomineralization within the triple membrane is fundamentally distinct from other well-investigated biomineralization processes which are either extracellular^54,67,68^ or intracellular^69^, respectively.

The presence of the organic matrix during crystal growth will also provoke the accumulation of organic materials on the growth surfaces^59–64^. This will eventually stop the growth of the crystal, and, as soon as supersaturation reaches the necessary threshold, nucleation of a secondary crystal in a twin-related orientation on the impurity-covered pre-existing crystal will occur. The described epitactic and homoepitactic effects related to nucleation and growth in the specific organic matrix apparently induces the formation of the newly discovered 78.2°|<9 9 1> and 76.6°|<6 −6 1> twin systems. If the latter were simply due to the common structural elements (coincidence site lattices) described in Tables 2 and 3, these orientation relationships would be expected in inorganic calcite as well. Future experiments need to show if in foraminiferal calcite the twin partners are in direct “inorganic” contact at all, or completely separated by organic sheaths. Indeed, although aragonite twinning in the nacre of *Mytilus edulis* (Linnaeus, 1758)^40,42^ follows the classical “pseudohexagonal” 64°|<001> = m.{110} twin law, by far the largest interface area between crystals in this twin orientation relationship is the aragonite {001} plane, rather than the {110} “twin-planes”. Following the classical purely inorganic/crystallographic perspective of twinning (coincidence site lattices), {001} would rather not be expected as contact plane for aragonite^40,42^. In nacre, the {001} surface planes and interface planes of the aragonite crystals are covered by the most prominent organic matrix sheets controlling the platy habit of the nacre aragonite. The orientation relationship, be it as a continuation of the growing crystal, or a continuation in twin orientation, is either mediated by the existing mineral bridges^70^ or by the organic matrix through an unknown templating mechanism which does not discriminate between the two twin orientations.

In summary, we showed that the analysis of crystal twinning in polycrystalline substances such as biologically formed calcite needs a proper mathematical treatment of three-dimensional orientation relationships and it benefits enormously from the statistical power of EBSD scanning experiments.

**(3) Thoughts on the biological role of micro- and nano-twinning**

The abundance of Ca^2+^ and (HCO_3_)^−^ in marine environments and the availability of physiological control mechanisms for their transport make Ca-carbonates a straightforward choice for reinforcement of tissues of marine life forms. However, calcite, aragonite and vaterite are highly fragile minerals, such that the organisms need to employ measures for mechanical strengthening of these minerals to gain evolutionary advantage. A general technique is to form hierarchical hybrid (organic-inorganic) composites. For anthropogenic engineered materials it is well known that nano-twinning increases strength and toughness^11–13^. Shin et al.^14^ and Li and Ortiz^15^ demonstrated that nano-twinning provides a toughening effect for Ca-carbonate shell materials as well. It may thus appear fair to hypothesize that the intense micro- and nano-twinning in foraminiferal calcite provides strengthening and toughening to the mineralized chamber walls, in particular for the delicate new bilamellar chamber walls of new chambers (while older chamber walls receive additional calcite layers when a new chamber is precipitated). The overview map of Fig. 1 indicates that the non-classical twinning schemes are concentrated at the dorsal side of the test, while the 60°|<001> twinning dominates in the central region of the ventral part, by which the *A. lessonii* attach to the substrate. This could hint at differences in the organic matrix composition in dorsal and ventral regions.

## Electronic supplementary material

Below is the link to the electronic supplementary material.

Supplementary Material 1

Supplementary Material 2

Supplementary Material 3

## Data Availability

The authors declare that the raw EBSD map data (positions and Euler angles) supporting the findings of this study are available within the supplementary information files.
