# Supplementary material for: Statistical analysis of EBSD data confirms pronounced classical and non-classical pervasive crystallographic twinning in rotaliid foraminiferal calcite
Source: Sci Rep. 2025 Apr 28;15:14852. doi: 10.1038/s41598-025-92636-y (PMC12037747; doi:10.1038/s41598-025-92636-y)
Supplement: Supplementary file 1 — Supplementary Material 1 [file 41598_2025_92636_MOESM1_ESM.pdf]

The file Euler_Angle_Data.txt contains the evaluated EBSD map as tab separated columns.

The table columns contain the following entries:

Serial number of data point, identified phase, x,y position of data point, three Euler angles obtained at this position (in ZXZ convention), comment about indexing.

A "-" in the second column means that no phase could be identified

A "-" in the last column indicates that indexing was not possible
