# Supplementary material for: Statistical analysis of EBSD data confirms pronounced classical and non-classical pervasive crystallographic twinning in rotaliid foraminiferal calcite
Source: Sci Rep. 2025 Apr 28;15:14852. doi: 10.1038/s41598-025-92636-y (PMC12037747; doi:10.1038/s41598-025-92636-y)
Supplement: Supplementary file 3 — Supplementary Material 3 [file 41598_2025_92636_MOESM3_ESM.docx]

APPENDIX

Proof of Eq. (16)

For a category II symmetry-equivalence between two orientation relationships (twin relationships) between domains A and B and A and C, respectively, we define Eq. 7 for the three domains, where the indices i, j, k designate the point group symmetry operators

$\mathbf{y}_{\mathrm{Ak}}=\left( \boldsymbol{\Omega}_{A}\mathbf{L}_{A} \right)\mathbf{S}_{k}\mathbf{x}_{1}$ (B1)

$$\mathbf{y}_{\mathrm{Bj}}=\left( \boldsymbol{\Omega}_{B}\mathbf{L}_{B} \right)\mathbf{S}_{j}\mathbf{x}_{1}$$

$$\mathbf{y}_{\mathrm{Ci}}=\left( \boldsymbol{\Omega}_{C}\mathbf{L}_{C} \right)\mathbf{S}_{i}\mathbf{x}_{1}$$

Likewise, with i, j, k =1 (the identity operation) Eq. 6 and 7 combine to

$\mathbf{y}_{B1}=\mathbf{R}_{\mathrm{AB}}\mathbf{y}_{A1}$ or $\mathbf{y}_{B1}=\left( \mathbf{R}_{\mathrm{AB}} \right)^{-1}\mathbf{y}_{B1}$ (B2a)

$\mathbf{y}_{C1}=\mathbf{R}_{\mathrm{AC}}\mathbf{y}_{A1}$ or $\mathbf{y}_{A1}=\left( \mathbf{R}_{\mathrm{AC}} \right)^{-1}\mathbf{y}_{C1}$ (B2b)

Likewise for B and C

$\mathbf{y}_{\mathrm{Ak}}=\left( \boldsymbol{\Omega}_{A}\mathbf{L}_{A} \right)\mathbf{S}_{k}\mathbf{x}_{1}$ (B3a)

$\mathbf{y}_{\mathrm{Bj}}=\left( \boldsymbol{\Omega}_{B}\mathbf{L}_{B} \right)\mathbf{S}_{j}\mathbf{x}_{1}$ (B3b)

$\mathbf{y}_{\mathrm{Ci}}=\left( \boldsymbol{\Omega}_{C}\mathbf{L}_{C} \right)\mathbf{S}_{i}\mathbf{x}_{1}$ (B3c)

The symmetry operations expressed in the joint Cartesian reference frame (Eq. 6 and 7) for the three differently oriented crystals (domains) become

$\mathbf{S}_{\mathrm{Ai}}^{O}={\left( \boldsymbol{\Omega}_{A}\mathbf{L}_{A} \right) \mathbf{S}}_{i} \left( \boldsymbol{\Omega}_{A}\mathbf{L}_{A} \right)^{-1}$ (B4a)

$\mathbf{S}_{\mathrm{Bi}}^{O}={\left( \boldsymbol{\Omega}_{B}\mathbf{L}_{B} \right) \mathbf{S}}_{i} \left( \boldsymbol{\Omega}_{B}\mathbf{L}_{B} \right)^{-1}$ (B4b)

$\mathbf{S}_{\mathrm{Ci}}^{O}={\left( \boldsymbol{\Omega}_{C}\mathbf{L}_{C} \right) \mathbf{S}}_{i} \left( \boldsymbol{\Omega}_{C}\mathbf{L}_{C} \right)^{-1}$ (B4c)

Note that for twin domains or crystals of the same phase, the three **L** matrices are identical. The orientation information is carried in the three matrices $\boldsymbol{\Omega}_{A,B, or C}$, respectively. We designate the transformation of coordinates in joint Cartesian reference space as

$\mathbf{y}_{\mathrm{Ak}}=\mathbf{S}_{\mathrm{Ak}}^{O}\mathbf{y}_{A1}$ (B5a)

$\mathbf{y}_{\mathrm{Bj}}=\mathbf{S}_{\mathrm{Bj}}^{O}\mathbf{y}_{B1}$ (B5b)

$\mathbf{y}_{\mathrm{Ci}}=\mathbf{S}_{\mathrm{Ci}}^{O}\mathbf{y}_{C1}$ (B5c)

To generate symmetry-equivalent misorientations (or twin laws) we first apply a symmetry-operation i of C onto C in the orientation of C, i.e. an internal relabeling of the crystallographic axes of C. With Eq. B5c and B2b we have

$\mathbf{y}_{\mathrm{Ci}}=\mathbf{S}_{\mathrm{Ci}}^{O}\mathbf{y}_{C1}=\mathbf{S}_{\mathrm{Ci}}^{O}\mathbf{R}_{\mathrm{AC}}\mathbf{y}_{A1}$ (B6)

Next, we apply symmetry-operation j of crystal A reorienting the whole crystal C_i_ :

$\mathbf{y}_{\mathrm{Cij}}=\mathbf{S}_{\mathrm{Aj}}^{o}\mathbf{y}_{\mathrm{Ci}}=\mathbf{S}_{\mathrm{Aj}}^{o}{\mathbf{S}_{\mathrm{Ci}}^{o}\mathbf{R}_{\mathrm{AC}}\mathbf{y}}_{A1}$ (B7)

Defining the symmetry-equivalent mapping from A to C as $\mathbf{R}_{\mathrm{ACij}}$

$\mathbf{y}_{\mathrm{ACij}}=\mathbf{R}_{\mathrm{ACij}}\mathbf{y}_{A1}$ (B8)

we finally have Eq. 16

$\mathbf{R}_{\mathrm{ACij}}=\mathbf{S}_{\mathrm{Aj}}^{o}{\mathbf{S}_{\mathrm{Ci}}^{o}\mathbf{R}_{\mathrm{AC}}}$ (B9)


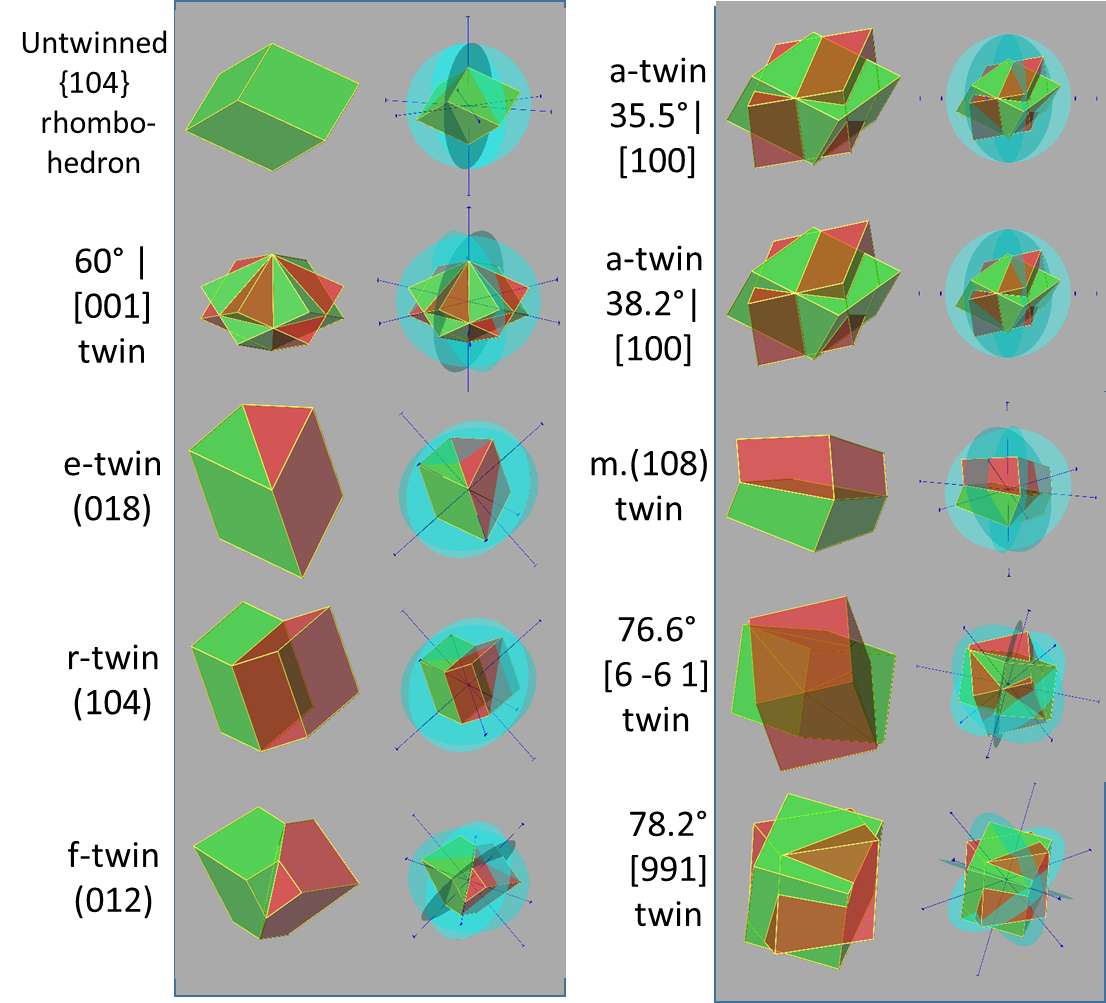


Fig. A1 Symbolic images of calcite twins listed in Table 2. For each twinning situation, one twin individual is shown in green, the other in red. The twins are shown with and without indication of symmetry elements of the „green“ individual. The e, r, and f twins are usually contact twins with a planar interface as they are generated by a shear deformation of the lattice; the {108} twin is also drawn as a contact twin. All other twins are penetration twins with irregular contact planes. The images were generated with the software „Shape“ (https://www.shapesoftware.com/).
